# The associations of sugar-sweetened, artificially sweetened and naturally sweet juices with all-cause mortality in 198,285 UK Biobank participants: a prospective cohort study

**DOI:** 10.1186/s12916-020-01554-5

**Published:** 2020-04-24

**Authors:** Jana J. Anderson, Stuart R. Gray, Paul Welsh, Daniel F. Mackay, Carlos A. Celis-Morales, Donald M. Lyall, John Forbes, Naveed Sattar, Jason M. R. Gill, Jill P. Pell

**Affiliations:** 1 Institute of Cardiovascular and Medical Sciences, University of Glasgow, Glasgow, G12 8TA UK; 2 Institute of Health and Wellbeing, University of Glasgow, Glasgow, G12 8RZ UK; 3 Health Research Institute, University of Limerick, Limerick, Ireland

**Keywords:** UK Biobank, Sugar, Diet, Mortality, Fruit juice

## Abstract

**Background:**

Recent efforts to address the obesity epidemic have focused on sugar consumption, especially sugar-sweetened beverages. However, sugar takes many forms, is only one contributor to overall energy consumption and is correlated with other health-related lifestyle factors. The objective was to investigate the associations with all-cause mortality of sugar- and artificially sweetened beverages and naturally sweet juices.

**Methods:**

Setting: UK Biobank, UK. Participants joined the UK Biobank study from 2006 to 2010 and were followed up until 2016; 198,285 men and women aged 40–69 years were eligible for this study (40% of the UK Biobank), of whom 3166 (1.6%) died over a mean of 7 years follow-up. Design: prospective population-based cohort study. Exposure variables: dietary consumption of sugar-sweetened beverages, artificially sweetened beverages, naturally sweet juices (100% fruit/vegetable juices) and total sugar intake, self-reported via 24-h dietary assessment tool completed between 2009 and 2012. Main outcome: all-cause mortality. Cox regression analyses were used to study the association between the daily intake of the above beverages and all-cause mortality. Models were adjusted for sociodemographic, economic, lifestyle and dietary confounders.

**Results:**

Total energy intake, total sugar intake and percentage of energy derived from sugar were comparable among participants who consumed > 2/day sugar-sweetened beverages and > 2/day fruit/vegetable juices (10,221 kJ/day versus 10,381 kJ/day; 183 g versus 190 g; 30.6% versus 31.0%). All-cause mortality was associated with total sugar intake (highest quintile adj. HR 1.28, 95% CI 1.06–1.55) and intake of sugar-sweetened beverages (> 2/day adj. HR 1.84, 95% CI 1.42–2.37) and remained so in sensitivity analyses. An association between artificially sweetened beverage intake and mortality did not persist after excluding deaths in the first 2 years of follow-up (landmark analysis) nor after excluding participants with recent weight loss. Furthermore, the inverse association between fruit/vegetable juice intake and mortality did not persist after additional adjustment for a diet quality score.

**Conclusions:**

Higher mortality is associated with sugar-sweetened beverages specifically. The lack of an adverse association with fruit/vegetable juices suggests that source of sugar may be important and the association with artificially sweetened beverage may reflect reverse causation.

## Background

In spite of health warnings, the consumption of sugar remains high worldwide. Dietary sugar takes different forms—solid or liquid, naturally occurring or added and intrinsic or extrinsic to cells—and different chemical compositions such as glucose or fructose. Public health campaigns and policies, such as the UK sugar tax, have specifically targeted sugar-sweetened beverages because they provide calories without producing satiety (hidden calories). It has been suggested that consumption of sugar-sweetened beverages may be associated with 184,000 deaths annually worldwide: 133,000 from diabetes, 45,000 from cardiovascular disease and 6450 from cancer [1]. Regular consumption increases total energy intake which in the absence of higher energy expenditure leads to obesity [2].

Few studies examined whether consumption of sugar-sweetened beverages is associated with all-cause mortality and, if so, whether the association is specific to this source of sugar and independent of overall sugar or energy consumption and adiposity. Also, conflicting results have been reported in the association with artificially sweetened drinks, which contain no sugar or calories. Study of two US cohorts, the Health Professional’s Follow-up Study and the Nurses’ Health study, found dose-response associations for sugar-sweetened beverages (SSB) intake, with increased risk starting for those who consume these drinks less than once a week compared to less than once a month [3]. However, they found that the consumption of artificially sweetened beverages (ASB) was only associated with all-cause mortality in women. Another study that examined the associations with the SSB and ASB intake in participants of the European Prospective Investigation into Cancer and Nutrition (EPIC) found an association between both SSB and ASB however only for those who consume large amounts (1 or more glasses daily) compared to less than once a month, with ASB showing a higher risk than SSB for the most frequent consumers [4]. ASB were also positively associated with all-cause mortality in the most frequent consumers (2 or more glasses daily) in the study of postmenopausal women in the USA [5]. With regard to naturally sweet juices (100% pure fruit or vegetable juices) and all-cause mortality, the evidence is scarce. An inverse association of 100% fruit juices and all-cause mortality was detected in the Japan Collaborative Cohort Study for Evaluation of Cancer [6]; however, a secondary analysis of data from the Reasons for Geographic and Racial Differences in Stroke (REGARDS) Study in the USA found a positive association between the intake of 100% fruit juices and all-cause mortality [7]. Therefore, more large prospective studies are required to examine the association with all-cause mortality by the source of sugar.

## Methods

The aim of this study was to compare consumption of total sugar, sugar-sweetened beverages, naturally sweet juices (100% fruit/vegetable juices) and artificially sweetened beverages with regard to their associations with all-cause mortality in a large, UK general population cohort.

### UK Biobank

Between April 2007 and December 2010, the UK Biobank recruited 502,682 participants aged 40–69 years from the general population. Participants attended one of 22 assessment centres across England, Wales and Scotland. They provided sociodemographic (age, ethnicity, income, highest qualification) and lifestyle (smoking status and sedentary behaviours) information via a self-completed, touch-screen questionnaire. Area-based socioeconomic status was determined from the postcode of residence using the Townsend index which is derived from census data on housing, employment, social class and car availability. Anthropometric measurements, including height, weight and waist circumference, were taken, at baseline, by trained nurses using standard operating procedures. Body mass index (BMI) was calculated as weight/height^2^.

Dietary information was collected via the Oxford WebQ, a web-based 24-h dietary assessment tool that was developed specifically for use in large population studies and has been validated against an interviewer-administered 24-h recall questionnaire [8]. Participants were invited to complete the online questionnaire on five occasions between April 2009 and June 2012. For participants who completed more than one questionnaire, we used the mean dietary intake. In our study after exclusions (see inclusion and exclusion criteria), 23% of participants completed two questionnaires, 21% three, 15% four and 3% five. Comparing dietary measures based on the first 24-h recall questionnaire completed versus the mean of all completed questionnaires, the Pearson correlation coefficients between the two measures were as follows: sugar-sweetened beverages, 0.802; artificially sweetened beverages, 0.852; fruit/vegetable juice, 0.835; and total sugar, 0.843. This correlation illustrates the association between the two possible measures used in the analyses.

In addition to food consumption, participants recorded how many glasses/cans of sugar- and artificially sweetened beverages (e.g. squash, cordial or fizzy drinks) and how many glasses/cartons/250 ml of pure fruit/vegetable juice they had drunk the previous day. Intake per day refers to these units. Total energy intake and total energy derived from sugar were calculated from the information recorded in the 5th edition of McCance and Widdowson’s “The composition of food”, and the percentage of total energy derived from sugar was derived. In a sensitivity analysis, a diet quality score was created by combining five dietary measures (mean intake of fat, fruit, vegetables, red meat and processed meat intake) according to the scoring system used in the Alternative Healthy Eating Index (AHEI) [9]. For each of the dietary groups, participants were grouped into tertiles of intake and scored 0, 5 and 10 respectively for the least to most healthy tertiles, most healthy being defined as low fat, sugar, red and processed meat, and high fruit or vegetable intake. The scores were summed producing an overall score ranging from 0 to 50. Physical activity undertaken over the previous 24 h was self-reported along with the Oxford WebQ, using questions based on the International Physical Activity Questionnaire (IPAQ). The duration of light, moderate and vigorous physical activity was converted into metabolic equivalents (MET-h/week) by applying weights of 2.5, 4 and 8, respectively, and then summed to derive an overall daily energy expenditure from physical activity.

Deaths were ascertained via linkage to the death certificates held by the National Health Service (NHS) Information Centre for England and Wales, and the NHS Central Register for Scotland. At the time of analysis, mortality data were available starting from baseline up to 31 January 2016.

### Inclusion and exclusion criteria

Inclusion was restricted to participants who had completed the online 24-h recall questionnaire on at least one occasion. We calculated basal metabolic rate using the Oxford equations [10] and excluded participants missing any information required to calculate it. We also excluded participants whose overall energy intake was suggestive of current dieting or under-reporting (defined as 500 kcal < 1.1 × basal metabolic rate) or over-reporting (defined as 500 kcal > 2.5 × basal metabolic rate).

### Study design and statistical analyses

This is a prospective population-based cohort study. Daily consumption of sugar-sweetened beverages, artificially sweetened beverages and 100% fruit/vegetable juice was categorised into 0, ≤ 1, > 1–2, or > 2 units daily (the number of units daily refers to intake recorded as the number of glasses/cans for sugar- and artificially sweetened beverages and the number of glasses/cartons/250 ml for 100% fruit/vegetable juices), and all forms of sugar (i.e. total sugar) were converted into quintiles. The characteristics of participants were compared according to their consumption using Kruskal-Wallis tests and Spearman rank tests. Univariate Cox proportional hazard models were used to examine the associations between sugar consumption or beverages and all-cause mortality. The models were adjusted incrementally for sociodemographic factors (age, sex, ethnic group); economic and lifestyle factors (income, qualifications, total physical activity, sedentary behaviour, smoking status, alcohol), body mass index and total energy intake, and potential dietary confounders (red meat, processed meat, fruit, vegetables, total fat, total fibre and total sugar intake; total sugar was not used when total sugar intake was the exposure of interest). We tested for statistical interactions with covariates and, where statistically significant, undertook subgroup analyses. We conducted a series of sensitivity analyses. To check for reverse causality; firstly, we re-ran the models excluding participants who lost weight in the year prior the recruitment, then we re-ran them adjusting or excluding for conditions associated with unintentional weight loss (defined as the history of myocardial infarction, heart failure, cancer, chronic obstructive pulmonary disease, emphysema, pulmonary fibrosis or rheumatoid arthritis). We then excluded the first 2 years of follow-up in a landmark analysis, and finally, in addition to using mean dietary intake based on multiple questionnaires, we ran the models using only the first questionnaire completed as this provided dietary data most proximal to the baseline data collection. To determine if any effect of sugar consumption was mediated via an effect on total energy consumption, we re-ran the models without adjusting for total energy intake as a covariate. To determine if any effect of sugar consumption was mediated via obesity, we re-ran the analysis stratified by BMI group and with and without adjusting for BMI. To test for residual confounding of dietary factors, we re-ran the models using a diet quality score instead of individual dietary confounders. To test for confounding by the other beverage categories, we re-run the models mutually adjusted (i.e. if sugar-sweetened beverages were the exposure of interest, the models were also adjusted for the intake of artificially sweetened beverages and fruit/vegetable juices). To test how well does one or repeated 24-h dietary recalls capture average diet, we run a subgroup analysis, splitting the participants into two groups, those who completed 1–2 (61%) and those who completed > 2 (39%) dietary questionnaires.

## Results

Of the 211,049 participants who completed at least one online diet questionnaire, 12,228 were excluded due to misreporting of their total energy intake and 526 because the basal metabolic rate could not be calculated. Of the remaining participants (198,285), 3166 (1.6%) died over a mean follow-up period of 7 years. The most common causes of mortality were any cancer (*n* = 2032), cardiovascular disease (*n* = 595) and respiratory diseases (*n* = 141). Comparison of baseline characteristics of included and excluded UK Biobank participants are presented in Additional file 1: Table S1. Participants that were included in the analysis differed from the rest of the UK Biobank cohort in being less deprived, having higher income and educational attainment and lower body weight, and less likely to currently smoke. They also spent less time watching television but more time on the computer.

Of the 198,285 study participants who all completed at least one online diet questionnaire, 65,027 (32.8%) drank sugar-sweetened beverages, 40,791 (20.6%) drank artificially sweetened beverages and 103,717 (52.2%) drank fruit/vegetable juice. Of those that drank these beverages, 51,842 (26.2%) participants drank ≤ 1 sugar-sweetened beverage daily, 9415 (4.8%) > 1–2 daily and 3770 (1.9%) > 2 daily; 27,079 (13.7%) drank ≤ 1 artificially sweetened beverage daily, 8860 (4.4%) > 1–2 daily and 5032 (2.5%) > 2 daily; and 89,206 (45.0%) drank ≤ 1 fruit/vegetable juice daily, 12,492 (6.3%) > 1–2 daily and 2019 (1.0%) > 2 daily. Compared with participants who did not consume sugar-sweetened beverages daily, those who did had a higher total energy intake, a higher total intake of sugar and a higher percentage of energy intake derived from sugar (Table 1). Participants who consumed fruit/vegetable juice also had a higher total energy intake, higher total intake of sugar and a higher percentage of energy intake derived from sugar. Total energy intake, total sugar intake and percentage of energy intake derived from sugar were all comparable among participants who consumed more than one sugar-sweetened beverage daily and those who consumed more than one fruit/vegetable juice daily. Consumption of artificially sweetened beverages was not associated with higher sugar consumption but slightly higher total energy intake, and higher BMI was found among those consuming > 2 artificially sweetened beverages daily.

**Table 1:** Energy intake by categories of beverage intake

	Total energy intake, mean (SD) (kJ/day)	Total sugar intake, mean (SD) (g/day)	Percentage of total energy intake derived from total sugar	Total sugar intake from sugar-sweetened beverages, mean (SD) (g/day)	Percentage of total energy intake derived from sugar-sweetened beverages
Sugar-sweetened beverages					
0	8658 (2273)	113.6 (44.0)	22.2	0	0
≤ 1	9078 (2202)	128.1 (42.7)	23.9	20.3 (10.5)	3.9
> 1–2	9734 (2483)	154.2 (48.6)	27.0	59.8 (10.6)	10.9
> 2	10,221 (2712)	183.0 (58.0)	30.6	116.4 (33.7)	20.2
Artificially sweetened beverages					
0	8858 (2299)	121.0 (46.1)	23.1	9.7 (21.9)	1.8
≤ 1	8791 (2198)	119.2 (43.5)	22.9	12.2 (19.6)	2.2
> 1–2	8826 (2441)	120.1 (48.9)	23.0	13.9 (24.8)	2.6
> 2	8914 (2570)	120.6 (55.5)	22.8	14.3 (29.8)	2.6
Fruit/vegetable juice					
0	8580 (2348)	110.1 (45.0)	21.7	10.5 (23.6)	2
≤ 1	9000 (2186)	126.0 (42.2)	23.7	10.0 (19.8)	1.8
> 1–2	9562 (2299)	150.7 (46.6)	26.8	11.2 (22.3)	1.9
> 2	10,381 (2708)	189.7 (70.4)	31	12.8 (30.0)	2

*SD* standard deviation

Consumption of sugar-sweetened and artificially sweetened beverages and fruit/vegetable juice differed in their associations with sociodemographic characteristics, lifestyle factors and other dietary factors except that high consumption of all three beverage types was associated with spending more time driving or at the computer (Table 2). Higher consumption of both sugar-sweetened and artificially sweetened beverages was associated with younger age, lower education levels, spending more time watching television, higher body mass indices and higher consumption of red and processed meat (all *p* < 0.01). Higher consumption of sugar-sweetened beverages was also associated with lower income and lower fruit and vegetable consumption. In contrast to higher consumers of artificially sweetened beverages who were more likely to be women and less physically active, higher consumers of sugar-sweetened beverages and fruit/vegetable juices were more likely to be men and physically active. Higher consumption of fruit/vegetable juice was associated with higher income, higher education levels, higher consumption of solid fruit and vegetable, spending less time watching television and lower consumption of red meat. Also, participants who consumed more fruit/vegetable juice did not have higher body mass indices in spite of higher total energy intake and higher intake of both sugar and fat (Tables 1 and 2).

**Table 2:** Characteristics of participants by categories of beverage intake

	Sugar-sweetened beverages				Artificially sweetened beverages				Fruit or vegetable juices			
	0/day, *N* = 133,258, mean (SD)	≤ 1/day, *N* = 51,842, mean (SD)	> 1–2/day, *N* = 9415, mean (SD)	> 2/day, *N* = 3770, mean (SD)	0/day, *N* = 157,494, mean (SD)	≤ 1/day, *N* = 27,079, mean (SD)	> 1–2/day, *N* = 8680, mean (SD)	> 2/day, *N* = 5032, mean (SD)	0/day, *N* = 94,568, mean (SD)	≤ 1/day, *N* = 89,206, mean (SD)	> 1–2/day, *N* = 12,492, mean (SD)	> 2/day, *N* = 2019, mean (SD)
Age (years)	56.5 (7.8)	55.8 (8.0)	54.0 (8.2)	52.7 (8.2)	56.6 (7.9)	54.9 (7.9)	53.8 (8.1)	52.9 (8.2)	55.9 (8.0)	56.5 (7.9)	55.8 (8.0)	55.2 (8.0)
Deprivation index	− 1.62 (2.8)	− 1.65 (2.8)	− 1.35 (3.0)	− 1.11 (3.1)	− 1.61 (2.9)	− 1.72 (2.8)	− 1.45 (2.9)	− 1.20 (3.0)	− 1.46 (2.9)	− 1.79 (2.8)	− 1.52 (2.9)	− 0.90 (3.2)
Driving (h/day)	0.9 (1.0)	0.9 (1.0)	1.0 (1.2)	1.1 (1.3)	0.9 (0.9)	0.9 (1.0)	1.0 (1.1)	1.1 (1.2)	0.9 (1.0)	0.9 (0.9)	0.9 (0.9)	1.0 (1.2)
Television (h/day)	2.5 (1.5)	2.5 (1.5)	2.6 (1.5)	2.8 (1.6)	2.5 (1.5)	2.6 (1.5)	2.8 (1.5)	3.0 (1.6)	2.7 (1.5)	2.4 (1.4)	2.3 (1.4)	2.3 (1.5)
Leisure time computer (h/day)	1.3 (1.3)	1.4 (1.3)	1.4 (1.4)	1.5 (1.5)	1.3 (1.3)	1.4 (1.3)	1.4 (1.3)	1.4 (1.5)	1.3 (1.3)	1.3 (1.3)	1.4 (1.4)	1.6 (1.5)
Body mass index	26.7 (4.5)	27.0 (4.5)	27.6 (4.9)	28.4 (5.4)	26.5 (4.3)	27.8 (4.8)	28.9 (5.2)	29.9 (5.9)	27.1 (4.7)	26.6 (4.4)	26.6 (4.3)	27.2 (4.8)
Waist circumference (cm)	88 (13)	89 (13)	91 (14)	94 (14)	88 (13)	90 (13)	93 (14)	95 (15)	89 (13)	88 (13)	89 (13)	91 (14)
Alcohol intake (g/day)	17 (22)	15 (19)	14 (21)	13 (22)	17 (22)	16 (19)	15 (21)	14 (21)	16 (22)	17 (20)	16 (21)	14 (22)
Total fat intake (g/day)	77 (28)	80 (27)	84 (30)	85 (33)	78 (28)	77 (26)	78 (30)	80 (32)	77 (23)	79 (27)	80 (28)	81 (33)
Fresh fruit (servings/day)	2.2 (2)	2.1 (2)	2.0 (2)	1.9 (2)	2.1 (2)	2.2 (2)	2.2 (2)	2.2 (2)	2.1 (2)	2.2 (2)	2.4 (2)	2.6 (2)
Vegetables (servings/day)	3.0 (3)	2.8 (3)	2.6 (3)	2.5 (3)	2.9 (3)	2.9 (3)	2.9 (3)	3.0 (3)	2.9 (3)	2.9 (3)	3.1 (3)	3.4 (3)
Red meat (servings/day)	0.3 (0.4)	0.3 (0.4)	0.3 (0.5)	0.4 (0.5)	0.3 (0.4)	0.3 (0.4)	0.3 (0.5)	0.4 (0.5)	0.3 (0.5)	0.3 (0.4)	0.3 (0.4)	0.3 (0.5)
Processed meat (servings/day)	0.5 (0.9)	0.6 (0.8)	0.6 (0.9)	0.7 (1.0)	0.5 (0.9)	0.6 (0.8)	0.6 (0.9)	0.7 (1.0)	0.5 (0.9)	0.6 (0.8)	0.6 (0.8)	0.5 (0.9)
Sugar-sweetened beverages (drinks/day)	0	0.6 (0.3)	1.7 (0.3)	3.3 (1.0)	0.3 (0.6)	0.4 (0.6)	0.4 (0.7)	0.4 (0.9)	0.3 (0.7)	0.3 (0.6)	0.3 (0.6)	0.4 (0.9)
Fruit/veg juices (drinks/day)	0.4 (0.6)	0.4 (0.6)	0.4 (0.6)	0.4 (0.6)	0.4 (0.6)	0.4 (0.5)	0.3 (0.5)	0.3 (0.6)	0	0.7 (0.3)	1.6 (0.3)	3.1 (0.9)
Artificially sweetened beverages (drinks/day)	0.3 (0.8)	0.3 (0.7)	0.4 (0.9)	0.4 (1.2)	0	0.6 (0.3)	1.8 (0.3)	4.0 (1.8)	0.3 (0.9)	0.2 (0.7)	0.2 (0.7)	0.2 (0.8)
	***N*(%)**	***N*(%)**	***N*(%)**	***N*(%)**	***N*(%)**	***N*(%)**	***N*(%)**	***N*(%)**	***N*(%)**	***N*(%)**	***N*(%)**	***N*(%)**
Male	56,288 (42)	23,794 (46)	5016 (53)	2221 (59)	70,904 (45)	11,002 (41)	3423 (39)	1990 (40)	39,121 (41)	40,705 (46)	6335 (51)	1158 (57)
White ethnicity	128,136 (97)	49,393 (96)	8839 (94)	3518 (94)	150,679 (96)	26,128 (97)	8271 (96)	4808 (96)	90,247 (96)	86,058 (97)	11,805 (95)	1776 (89)
Income < £18,000 pa	18,311 (15)	6933 (15)	1419 (17)	638 (19)	21,996 (16)	3353 (14)	1182 (15)	770 (17)	14,862 (18)	10,698 (13)	1460 (13)	281 (15)
Degree	58,036 (48)	22,273 (47)	3599 (42)	1261 (37)	69,263 (48)	11,007 (44)	3187 (41)	1712 (38)	34,918 (42)	42,399 (51)	6756 (58)	1096 (58)
Current smoker	10,211 (8)	3715 (7)	895 (10)	417 (11)	12,151 (8)	1870 (7)	712 (8)	505 (10)	8672 (9)	5512 (6)	898 (7)	156 (8)
Physical activity ≥ 600 MET/day	52,287 (39)	20,919 (40)	4027 (43)	1659 (44)	62,577 (40)	10,925 (40)	3443 (40)	1947 (39)	37,207 (39)	35,546 (40)	5273 (42)	866 (43)

*SD* standard deviation, *N* number, *h* hours; pa per annum, *MET* metabolic equivalents

All *p* < 0.010

In the Cox proportional hazard models, the highest quintile of total sugar consumption was associated with all-cause mortality after adjustment for socioeconomic and lifestyle confounders and total fat intake, and after additional adjustment for fruit, vegetables, total fibre and meat intake (Table 3). The association was still apparent when the first 2-years of follow-up were excluded (the landmark analysis, Additional file 2: Table S2b) but not when total sugar consumption was based on only the first food questionnaire completed following recruitment (Additional file 3: Table S3b).

**Table 3:** Cox proportional hazard model of the association between total sugar consumption and all-cause mortality

	Number	Deaths, no.	First quintile, 0.4–81.9 g, *n* = 39,660, HR (95% CI)	Second quintile, 82–105 g, *n* = 39,655, HR (95% CI)	Third quintile, 106–126 g, *n* = 39,663, HR (95% CI)	Fourth quintile, 127–154 g, *n* = 39,650, HR (95% CI)	Highest quintile, 155–1051 g, *n* = 39,657, HR (95% CI)	*p* value for trend
Deaths, no.			632	615	599	599	723	
Model								
0	198,283	3166	1 (Ref)	0.96 (0.86–1.08)	0.94 (0.84–1.05)	0.94 (0.84–1.05)	1.15 (1.03–1.27)	0.034
1	197,590	3150	1 (Ref)	0.90 (0.81–1.01)	0.84 (0.75–0.94)	0.81 (0.73–0.91)	0.95 (0.86–1.06)	0.156
2	161,415	2311	1 (Ref)	1.02 (0.97–1.27)	0.98 (0.87–1.15)	1.00 (0.94–1.25)	1.19 (0.95–1.30)	0.108
3	161,415	2311	1 (Ref)	1.04 (0.91–1.19)	0.99 (0.87–1.15)	1.03 (0.88–1.20)	1.25 (1.04–1.50)	0.076
4	161,415	2311	1 (Ref)	1.05 (0.92–1.20)	1.02 (0.88–1.18)	1.05 (0.90–1.23)	1.28 (1.06–1.55)	0.057

Model 0: unadjusted

Model 1: adjusted for sex, age and ethnicity

Model 2: model 1 also adjusted for income, highest qualification, physical activity, sedentary behaviour, total energy intake, body mass index, smoking status and alcohol intake

Model 3: model 2 also adjusted for total fat intake

Model 4: model 3 also adjusted for fresh fruit intake, vegetable intake, total fibre intake, red meat intake and processed meat intake

Median total sugar intake: 1st quintile, 68 g; 2nd quintile, 94 g; 3rd quintile, 115 g; 4th quintile, 139 g; 5th quintile, 179 g

*N* number, *HR* hazard ratio, *CI* confidence interval

There was evidence of a dose-response relationship between consumption of sugar-sweetened beverages and all-cause mortality (most adjusted model HR trend 1.16 (95% CI 1.09–1.23, *p* < 0.001) that persisted following adjustment for potential confounders, including total sugar intake and other dietary components (Table 4). In the sensitivity analyses, the association between sugar-sweetened beverage consumption and mortality persisted following adjustment for consumption of artificially sweetened beverages and fruit/vegetable juices (Additional file 4: Table S4) and adjustment for a diet quality score (Additional file 5: Table S5) and was apparent whether or not total energy intake was included as a covariate (Additional file 6: Table S6a). However, when stratified by BMI group, there was no association in participants with normal BMI (Additional file 7: Table S7). When BMI was excluded from the multivariate model, there was a slight increase in the association (Additional file 6: Table S6b). The association also persisted when a landmark analysis was conducted (Additional file 2: Table S2a) and when we adjusted for participants with a prevalent disease at baseline (Additional file 8: Table S8). When these participants were excluded from the analysis, the association was lost for those consuming > 1–2 beverages daily but remained for those consuming > 2 beverages daily (Additional file 8: Table S8). Similar results were found when the intake of sugar-sweetened beverages was derived from only the first dietary questionnaire completed following recruitment (Additional file 3: Table S3a). When the analysis was stratified by the number of dietary questionnaire completed (1–2 versus > 2), the association between sugar-sweetened beverage consumption and mortality for those who completed > 2 questionnaires persisted only in the > 2/day category of intake (Additional file 9: Table S9). Additional stratified analysis by all the covariates included in the most adjusted model is presented in Additional file 11: Table S11.

**Table 4:** Cox proportional hazards models of the associations between categories of beverage intake and all-cause mortality

**Sugar-sweetened beverages**							
**Model**	**Number**	**Deaths, no.**	**0/day,*n*= 133,258,HR (95% CI)**	**1/day,*n*= 51,842,HR (95% CI)**	**> 1–2/day,*n*= 9415,HR (95% CI)**	**> 2/day,*n*= 3770,HR (95% CI)**	***p*value for trend**
Deaths, no.			2074	834	160	98	
0	198,283	3166	1 (Ref)	1.03 (0.95–1.12)	1.10 (0.94–1.30)	1.71 (1.40–2.09)	< 0.001
1	197,590	3150	1 (Ref)	1.07 (0.98–1.16)	1.28 (1.09–1.51)	2.13 (1.74–2.62)	< 0.001
2	161,415	2311	1 (Ref)	1.06 (0.97–1.17)	1.35 (1.12–1.62)	1.86 (1.44–2.40)	< 0.001
3	161,415	2311	1 (Ref)	1.06 (0.97–1.17)	1.35 (1.12–1.63)	1.87 (1.45–2.42)	< 0.001
4	161,415	2311	1 (Ref)	1.06 (0.96–1.16)	1.33 (1.10–1.60)	1.84 (1.42–2.37)	< 0.001
**Artificially sweetened beverages**							
**Model**	**Number**	**Deaths, no.**	**0/day,*n*= 157,494,HR (95% CI)**	**1/day,*n*= 27,079,HR (95% CI)**	**> 1–2/day,*n*= 8680,HR (95% CI)**	**> 2/day,*n*= 5032,HR (95% CI)**	***p*value for trend**
Deaths, no.			2550	377	144	95	
0	198,283	3166	1 (Ref)	0.84 (0.75–0.93)	1.06 (0.90–1.26)	1.24 (1.01–1.52)	0.743
1	197,590	3150	1 (Ref)	0.98 (0.88–1.09)	1.35 (1.14–1.60)	1.73 (1.41–2.12)	< 0.001
2	161,415	2311	1 (Ref)	0.92 (0.81–1.05)	1.13 (0.91–1.39)	1.44 (1.12–1.84)	0.063
3	161,415	2311	1 (Ref)	0.92 (0.81–1.05)	1.13 (0.91–1.39)	1.44 (1.12–1.84)	0.058
4	161,415	2311	1 (Ref)	0.92 (0.81–1.05)	1.13 (0.91–1.39)	1.44 (1.12–1.84)	0.062
**Fruit or vegetable juice**							
**Model**	**Number**	**Deaths, no.**	**0/day,*n*= 94,568,HR (95% CI)**	**1/day,*n*= 89,206,HR (95% CI)**	**> 1–2/day,*n*= 12,492,HR (95% CI)**	**> 2/day,*n*= 2019,HR (95% CI)**	***p*value for trend**
Deaths, no.			1598	1362	181	25	
0	198,283	3166	1 (Ref)	0.89 (0.83–0.95)	0.84 (0.72–0.98)	0.73 (0.49–1.08)	> 0.001
1	197,590	3150	1 (Ref)	0.82 (0.77–0.89)	0.80 (0.67–0.94)	0.76 (0.49–1.08)	> 0.001
2	161,415	2311	1 (Ref)	0.91 (0.83–0.99)	0.89 (0.74–1.06)	0.64 (0.39–1.05)	0.008
3	161,415	2311	1 (Ref)	0.89 (0.82–0.97)	0.84 (0.70–1.01)	0.58 (0.35–0.96)	0.001
4	161,415	2311	1 (Ref)	0.89 (0.81–0.97)	0.83 (0.69–1.00)	0.57 (0.34–0.93)	0.001

Model 0: unadjusted

Model 1: adjusted for sex, age and ethnicity

Model 2: model 1 also adjusted for income, highest qualification, physical activity, sedentary behaviour, total energy intake, body mass index, smoking status and alcohol intake

Model 3: model 2 also adjusted for total sugar intake and total fat intake (total sugar intake was not included in the analysis of sugar-sweetened beverages)

Model 4: model 3 also adjusted for fresh fruit intake, vegetable intake, total fibre intake, red meat intake and processed meat intake

*N* number, *HR* hazard ratio, *CI* confidence interval

Consumption of fruit/vegetable juice demonstrated an apparent inverse dose-response relationship with all-cause mortality (Table 4), the most adjusted model HR for trend 0.89 (95% CI 0.83–0.95, *p* = 0.001). The association was present irrespective of whether total energy was included as a covariate (Additional file 6: Table S6a) and persisted in the highest category when dietary intake was based on only the first questionnaire completed (Additional file 3: Table S3a). There were significant interactions between the intake of fruit/vegetable juices and age (*p* < 0.001) and physical activity (*p* = 0.005). The inverse association was only detected for those > 55 years of age (> 2 fruit/vegetable juices fully adjusted HR 0.53, 95% CI 0.30–0.95, *p* = 0.032) and for the least physically active (> 1–2 fruit/vegetable juice fully adjusted HR 0.59, 95% CI 0.39–0.90, *p* = 0.015). The protective association with fruit/vegetable juice consumption was still observed after adjusting for consumption of sugar- and artificially sweetened beverages (Additional file 4: Table S4) but not after adjusting for a diet quality score (Additional file 5: Table S5). Also, whilst it persisted following adjustment for prevalent disease at baseline (Additional file 8: Table S8) and in the landmark analysis (Additional file 2: Table S2a), it was no longer observed in the highest intake category when participants with these conditions were excluded from the analysis (Additional file 8: Table S8), for any intake for those who completed > 2 dietary questionnaires (Additional file 9: Table S9) or when stratified by BMI group, except for obese participants drinking up to 1 fruit/vegetable juice daily (Additional file 7: Table S7). Excluding BMI from the multivariate model had no effect on the association (Additional file 6: Table S6b). Additional stratified analysis by all the covariates included in the most adjusted model is presented in Additional file 11: Table S11.

Daily consumption of artificially sweetened beverages was not associated with higher all-cause mortality in the overall trend, most adjusted model HR for trend 1.06 (95% CI 1.00–1.14, *p* = 0.062), and only the consumption of > 2 artificially sweetened beverages daily was significant (Table 4). The association had significant interactions with sex (*p* = 0.022), age (*p* = 0.003), ethnicity (*p* < 0.001), income (*p* = 0.009) and highest qualification (*p* = 0.006). The association was only observed in men (fully adjusted model: HR 1.93, 95% CI 1.43–2.60, *p* < 0.001) and among those over 55 years of age (fully adjusted model: HR 1.46, 95% CI 1.10–1.94, *p* = 0.008). It only reached significance among those on annual incomes of £31k–52k (fully adjusted model: HR 1.65, 95% CI 1.05–2.61, *p* = 0.031) and with the highest qualification level (fully adjusted model: HR 1.98, 95% CI 1.41–2.78, *p* < 0.001). The association persisted following adjustment for consumption of sugar-sweetened beverages and fruit/vegetable juices (Additional file 4: Table S4) and a diet quality score (Additional file 5: Table S5) and was present irrespective of whether total energy was included as a covariate (Additional file 6: Table S6a). Whilst it persisted following adjustment for the presence of prevalent disease at baseline and was stronger when participants with these conditions were excluded from the analysis (Additional file 8: Table S8), or when BMI was excluded from the multivariate model (Additional file 6: Table S6b), it was no longer significant after exclusion of those who had experienced weight loss in the previous year (Additional file 10: Table S10), in landmark analysis (Additional file 2: Table S2a), or stratified by BMI group, except for overweight participants consuming > 2 beverages daily (Additional file 7: Table S7) and for those who completed > 2 dietary questionnaires (Additional file 9: Table S9). In contrast, when intake was based on only the first questionnaire completed (Additional file 3: Table S3a), the association was detected for any intake over > 1 beverage daily. Additional stratified analysis by all the covariates included in the most adjusted model is presented in Additional file 11: Table S11.

## Discussion

Consumption of sugar-sweetened beverages was associated with all-cause mortality independent of other aspects of diet, including other beverages, and total energy consumption. Total sugar consumption was also associated with mortality but only for the highest quintile of consumption (over 155 g/day), where participants consumed higher amounts of sugar than the amounts of sugar consumed by the participants in the ≤ 1 and > 1–2 sugar-sweetened beverages/day category. Also, the association with sugar-sweetened beverages was independent of the total amount of sugar. Participants who drank 100% fruit/vegetable juice were comparable to participants who drank sugar-sweetened beverages, in terms of total sugar intake, total energy intake and percentage of energy derived from sugar, but were not at increased risk of mortality. These findings suggest that the risk associated with sugar-sweetened beverages may reflect the nature and delivery of sugar, rather than simply the amount. Liquid sugar may be more rapidly consumed and absorbed than solid sugar, or the other beneficial compounds and nutrients such as vitamins or fibre present in solid foods and fruit/vegetable juices may offset the adverse effects of sugar and confer some protection against these [11]. However, the intake of dietary fibre was adjusted for in our study. Whilst consumption of artificially sweetened beverages initially appeared to be associated with a higher risk of mortality in the highest intake category, sensitivity analyses suggested that this may be explained by reverse causation.

### Comparison with previous studies

Previous studies on sugar and mortality have produced conflicting results due, in part, to different approaches to measuring sugar intake including total sugar, percentage of energy derived from sugar and added or free sugar. Whilst some studies have suggested an increased risk associated with added sugar [12], others have not [13] or they have found negligible differences in the positive association between both added and free sugars [14]. A number of studies have focused on sugar-sweetened beverages. Consumers of sugar-sweetened beverages have worse cardiometabolic risk profiles in terms of inflammatory markers and glycaemia [11] and a higher risk of metabolic syndrome [15] after adjusting for body mass index and total energy intake. Meta-analyses, of five, ten and seven studies, respectively, suggest that consumption of sugar-sweetened beverages is associated with hypertension [16], type 2 diabetes [17] and stroke [18]. A meta-analysis of four studies suggested that sugar-sweetened beverages were associated with coronary heart disease [19]. Three of the Bradford Hill criteria for causation were fully met with some evidence in support of the other six criteria [20]. In contrast, a meta-analysis of two studies found no association with heart failure [18]. An association with all-cause mortality has been suggested by a meta-analysis of two studies from the USA but not when all five relevant studies were included [21]. Our contradictory finding of an association between sugar-sweetened beverages and all-cause mortality may be due to the higher cut-offs in our study than in four of the five studies included in the meta-analysis [13, 22–24]. Also, one of the studies in the meta-analysis included fruit/vegetable juice as a sugar-sweetened beverage [13]. When we re-ran our analyses using this approach, there was no association between intake of sugar-sweetened beverages (including fruit/vegetable juices) and all-cause mortality. Our findings however corroborate recent work from both European and US cohorts. A recently published study from 10 European countries found a positive association between both sugar-sweetened (1–2/day and > 2/day) and artificially sweetened beverages (> 2/day) and all-cause mortality when compared to referent category (< 1/month). This study used data on 451,743 participants over a mean follow-up of 16.4 years [25]. Another study used data from two US cohorts—the Nurses’ Health Study (NHS) of 80, 647 women and the Health Professionals Follow-up Study (HPFS) of 37,716 men [3]—and looked at the intake of sugar-sweetened and artificially sweetened beverages and all-cause mortality and mortality from cancers and cardiovascular causes over a follow-up of 34 and 28 years, respectively. In this study, any consumption of sugar-sweetened beverages associated with increased all-cause mortality (< 1–4/month, 2–6/week, 1–2/day and > 2/day) when compared to referent category (< 1/month) in women and 2–6/week and above in men.

It has been recommended that high consumption of artificially sweetened beverages should be discouraged [3] and not be promoted as a safe substitute for sugar-sweetened beverages [26]. Meta-analyses, of three and four studies, respectively, have demonstrated an association between consumption of artificially sweetened beverages and hypertension [27] and cardiovascular disease [4]. A meta-analysis of 17 studies showed that artificially sweetened beverages were also associated with type 2 diabetes, independent of adiposity [28]. Individual studies have demonstrated associations with metabolic syndrome [15], stroke [29] and all-cause mortality [30]. However, in the EPIC, NHS and HPFS cohorts, and in the Women’s Health Initiative Observational Study, only the highest intake of artificially sweetened beverages was associated with all-cause mortality [3–5], and a more recent systemic review and meta-analysis of randomised and non-randomised control trials and observational studies, of non-sugar sweeteners (including artificially sweetened beverages) was inconclusive in relation to whether they were associated with harm [31]. Our initial findings also suggested an association between artificially sweetened beverages and mortality in the highest intake category. However, further scrutiny suggested that the association was no longer statistically significant following exclusion of participants with recent weight loss and on landmark analysis, suggesting the findings may simply reflect reverse causation. It was also no longer statistically significant in those participants who completed > 2 dietary questionnaires. However, it was stronger when the participants with prevalent diseases at baseline were excluded from the analysis. The association was also stronger when BMI was not adjusted for in the multivariate model suggesting some of the effects might be mediated by adiposity. Therefore, further research is required.

Previous meta-analyses have consistently shown that, in spite of high sugar content, fruits are associated with reduced risk of many adverse health outcomes including hypertension [16], coronary heart disease [18, 20], stroke [18, 20, 32] and all-cause mortality [21, 32] but has a U-shaped association with type 2 diabetes [17]. However, few studies have examined fruit juice in relation to mortality [6, 7, 32, 33]. Our sensitivity analysis shows that findings on fruit/vegetable juices and all-cause mortality are sensitive to possible residual confounding and reverse causality (Additional files, Supplementary Tables) and as such require further research.

### Strengths and limitations of this study

The UK Biobank is a very large, prospective, general population cohort. Whilst UK Biobank participants are not representative of the general population (and hence cannot be used to provide representative disease prevalence and incidence rates), valid assessment of exposure-disease relationships is nonetheless widely generalizable and does not require participants to be representative of the population at large [34]. This study population is reasonably representative of the general population in terms of age, sex, ethnicity and socioeconomic breakdown, but is unrepresentative in terms of lifestyle. Therefore, caution should be heeded in generalising summary statistics, such as the distributions of sugar and beverage intake and absolute mortality. However, effect size estimates should, nonetheless, be generalizable. According to the recent meta-analysis, sugar-sweetened beverages are consumed by 49.4% of the UK population [28] whilst for the UK Biobank population included in the analysis, this was 32.8%. On the other hand, data from the 2008–2012 and 2013–2014 UK National Diet and Nutrition Surveys (NDNS) showed that artificially sweetened beverages are consumed by 17–19.8% of British subjects aged 16 and over [35], and the consumption in the included UK Biobank population was 20.6%. This difference could be due to the 4 days food record rather than the 24-h recall used in these studies [28, 35].

Some previous studies have combined the different categories of beverage included in our study such as sugar- and artificially sweetened beverages [36], thereby masking important differences, or split categories according to their caffeine content [23]. Although we were able to separate drinks by the type of sugar, we could not distinguish between different type of sweeteners used or exclude caffeine-containing drinks.

In our study, dietary information was obtained via repeated 24-h recall questionnaires where available which have been shown to be more accurate than food frequency questionnaires for commonly consumed foods [37]. We used the data from all completed questionnaires to correct for within-person variation in diet. However, the fact that 38% of participants only completed one 24-h dietary recall in the UK Biobank is a limitation. Single 24-h dietary recall method is limited in its ability to capture the consumption level among people who drank these beverages less than daily. Indeed, stratifying the analysis by the number of diet questionnaires filled (1–2 versus > 2) showed that the positive or negative associations for all-cause mortality and artificially sweetened beverages and fruit/vegetable juice intake, respectively, are only significant for those that completed 1–2 diet questionnaires, whilst these associations are no longer statistically significant for those that completed more than 2. However, we also undertook a sensitivity analysis using data from the first questionnaire completed as this was undertaken closer in time to recruitment when the information on confounders was collected, and this produced largely consistent findings. Since diet was based on self-report, reporting bias is plausible. We attempted to reduce the risk of this by excluding participants with implausible energy intake.

In any observational study, association does not necessarily imply causation, even in the presence of a dose-response relationship. We were able to adjust for a wide range of sociodemographic, lifestyle and dietary confounders. Whilst dietary and physical activity information was recorded together with exposure of interest, other confounders included in the analysis (such as smoking, sedentary behaviour and BMI) were only recorded at baseline and could have changed during the follow-up. However, residual confounding is still possible. This is most apparent in the sensitivity analysis of fruit/vegetable juices. In a sensitivity analysis, we adjusted for participants with a prevalent disease at baseline in order to reduce the risk of reverse causation, but it remains possible especially in relation to artificially sweetened beverages being consumed as part of a weight-reduction diet. We endeavoured to reduce the risk of this by excluding participants whose total energy intake was suggestive of a weight-reduction diet. To explore this further, we excluded those who reported weight loss in the 12 months prior to the recruitment, and the findings were reversed, where an inverse effect was detected for up to 1 drink daily whilst the increased risk for those consuming more than 1 drink/day was no longer significant. There was also no association between all-cause mortality and artificially sweetened beverages in the landmark analysis.

## Conclusions

Our study demonstrated that consumption of sugar-sweetened beverages was associated with all-cause mortality. However, this could not be attributed to their sugar-content alone since the association was independent of total sugar intake and total energy intake, and fruit/vegetable juice consumption was not associated with a higher risk of death in spite of similar levels of sugar and total energy intake. Artificially sweetened beverages also initially appeared to be associated with all-cause mortality in the highest intake category; however, this was sensitive to adjustment for reverse causation and residual confounding.

The type and number of beverages consumed were significantly associated with a wide range of sociodemographic, lifestyle and other dietary factors. Whilst the associations were independent of the measured confounders, it is highly likely that there are residual unknown or unmeasured confounder factors, and further research is needed.

## Supplementary information

**Additional file 1:Supplementary Table 1.** Comparison of baseline characteristics of included and excluded UK Biobank participants.

**Additional file 2:Supplementary Table 2a.**. Cox proportional hazards models of the associations between categories of beverage intake and all-cause mortality - Landmark analysis. **Supplementary Table 2b.** Cox proportional hazard model of the association between total sugar consumption and all-cause mortality – Landmark.

**Additional file 3:Supplementary Table 3a.** Cox proportional hazards models of the associations between categories of beverage intake and all-cause mortality -first intake. **Supplementary Table 3b.** Cox proportional hazard model of the association between total sugar consumption and all-cause mortality (first intake).

**Additional file 4:Supplementary Table 4.** Cox proportional hazards model4 of the associations between categories of beverage intake and all-cause mortality with or without mutual adjustment for other beverages.

**Additional file 5:Supplementary Table 5.** Cox proportional hazards models of the associations between categories of beverage intake and all-cause mortality –replacing Model 3 and model 4 with a diet quality score (score of processed meat, red meat, fruit, vegetable, fat and sugar intake).

**Additional file 6:Supplementary Table 6.a.** Cox proportional hazards model4 of the associations between categories of beverage intake and all-cause mortality with or without adjustment for total energy. **Supplementary Table 6.b.** Cox proportional hazards model4 of the associations between categories of beverage intake and all-cause mortality with or without adjustment for BMI.

**Additional file 7:Supplementary Table 7.** Cox proportional hazards -Model 4- of the associations between categories of beverage intake and all-cause mortality stratified by BMI group.

**Additional file 8:Supplementary Table 8.** Cox proportional hazards models of the associations between categories of beverage intake and all-cause mortality with added Model 5 –adjusted for prevalent disease at baseline (cancer *N* = 15,121), other conditions (*N* = 6333), Model 6 -the same conditions excluded instead.

**Additional file 9:Supplementary Table 9.** Cox proportional hazards -Model 4- of the associations between categories of beverage intake and all-cause mortality stratified by the number of complete dietary questionnaires (1–2 versus > 2).

**Additional file 10:Supplementary Table 10.** Cox proportional hazard model of the association between artificially-sweetened drinks and all-cause mortality, excluding those with weight loss one year prior baseline recruitment or missing information (*N* = 31,642).

**Additional file 11:Supplementary Table 11.** Cox proportional hazards -Model 4- of the associations between categories of beverage intake and all-cause mortality stratified by all covariates.

## Data Availability

The UK Biobank is an open-access resource, and all bona fide researchers can use it for approved research by registering and applying at http://www.ukbiobank.ac.uk/register-apply/. This research has been conducted using the UK Biobank Resource under Application Number 7155.

## Abbreviations

BMI: Body mass index
CI: Confidence interval
EPIC: European Prospective Investigation into Cancer and Nutrition
HR: Hazard ratio
MET: Metabolic equivalents
NHS: Nurses’ Health Study
HPFS: Health Professionals Follow-up Study
SD: Standard deviation
