# Supplementary material for: The associations of sugar-sweetened, artificially sweetened and naturally sweet juices with all-cause mortality in 198,285 UK Biobank participants: a prospective cohort study
Source: BMC Med. 2020 Apr 24;18:97. doi: 10.1186/s12916-020-01554-5 (PMC7181499; doi:10.1186/s12916-020-01554-5)
Supplement: Supplementary file 1 — Additional file 1:Supplementary Table 1. Comparison of baseline characteristics of included and excluded UK Biobank participants. [file 12916_2020_1554_MOESM1_ESM.docx]

Supplementary Table 1. Comparison of baseline characteristics of included and excluded UK Biobank participants

|  | Included, N=198,285 | Excluded, N=304,335 |
| --- | --- | --- |
|  | Died, N=3,166 | Died, N=11,254 |
|  |  |  |
|  |  |  |
|  | *Mean (SD)* | *Mean (SD)* |
| Age (years) | 56.1 (7.9) | 56.8 (8.2) |
| Deprivation index | -1.61 (2.9) | -1.09 (3.2) |
| Driving (h/day) | 0.9 (1.0) | 1.0 (1.3) |
| Television (h/day) | 2.5 (1.5) | 3.0 (1.6) |
| Computer (h/day) | 1.3 (1.3) | 0.9 (1.3) |
| Body mass index | 26.8 (4.5) | 27.8 (4.9) |
| Waist circumference (cm) | 89 (13) | 91 (13) |
|  | N (%) | N (%) |
| Male | 87,319 (44) | 141,841 (47) |
| White ethnicity | 189,886 (96) | 282,902 (94) |
| Income <£18,000 pa | 27,301 (15) | 69,912 (28) |
| Degree | 85,169 (47) | 76,030 (34) |
| Current smoker | 15,238  (7.7) | 37,748  (12.5) |
