# Supplementary material for: The associations of sugar-sweetened, artificially sweetened and naturally sweet juices with all-cause mortality in 198,285 UK Biobank participants: a prospective cohort study
Source: BMC Med. 2020 Apr 24;18:97. doi: 10.1186/s12916-020-01554-5 (PMC7181499; doi:10.1186/s12916-020-01554-5)
Supplement: Supplementary file 2 — Additional file 2:Supplementary Table 2a.. Cox proportional hazards models of the associations between categories of beverage intake and all-cause mortality - Landmark analysis. Supplementary Table 2b. Cox proportional hazard model of the association between total sugar consumption and all-cause mortality – Landmark. [file 12916_2020_1554_MOESM2_ESM.docx]

Supplementary table 2a. Cox proportional hazards models of the associations between categories of beverage intake and all-cause mortality - Landmark analysis

|  |  | |  |  | |  |  | |  |
| --- | --- | --- | --- | --- | --- | --- | --- | --- | --- |
|  | Sugar-sweetened beverages | |  | Artificially-sweetened beverages | |  | Fruit or vegetable juice | |  |
| Model | 1/day | >1-2/day | >2/day | 1/day | >1-2/day | >2/day | 1/day | >1-2/day | >2/day |
|  | n=51,842 | n=9,415 | n=3,770 | n=27,079 | n=8,680 | n=5,032 | n=89,206 | n=12,492 | n=2,019 |
|  |  |  |  |  |  |  |  |  |  |
|  | HR (95% CI) | HR (95% CI) | HR (95% CI) | HR (95% CI) | HR (95% CI) | HR (95% CI) | HR (95% CI) | HR (95% CI) | HR (95% CI) |
|  |  |  |  |  |  |  |  |  |  |
|  |  |  |  |  |  |  |  |  |  |
| 0 | 1.09 (1.00-1.18) | 1.11 (0.94-1.31) | 1.64 (1.31-2.03) | 0.90 (0.81-1.01) | 1.03 (0.86-1.24) | 1.13 (0.91-1.42) | 0.91 (0.85-0.99) | 0.87 (0.74-1.02) | 0.75 (0.50-1.13) |
| 1 | 1.12 (1.03-1.22) | 1.29 (1.09-1.53) | 2.05 (1.65-2.56) | 1.05 (0.94-1.18) | 1.32 (1.10-1.58) | 1.59 (1.27-1.99) | 0.85 (0.78-0.91) | 0.83 (0.70-0.97) | 0.75 (0.49-1.13) |
| 2 | 1.12 (1.02-1.24) | 1.35 (1.11-1.62) | 1.85 (1.41-2.41) | 0.99 (0.86-1.12) | 1.07 (0.86-1.35) | 1.27 (0.97-1.67) | 0.92 (0.85-1.02) | 0.91 (0.76-1.10) | 0.67 (0.40-1.11) |
| 3 | 1.13 (1.02-1.24) | 1.36 (1.12-1.65) | 1.87 (1.43-2.45) | 0.99 (0.87-1.13) | 1.08 (0.88-1.35) | 1.28 (0.97-1.68) | 0.92 (0.83-1.00) | 0.87 (0.72-1.05) | 0.61 (0.36-1.02) |
| 4 | 1.12 (1.01-1.24) | 1.34 (1.10-1.63) | 1.84 (1.40-2.41) | 0.99 (0.87-1.13) | 1.07 (0.86-1.35) | 1.27 (0.97-1.68) | 0.91 (0.83-0.99) | 0.86 (0.71-1.03) | 0.60 (0.36-1.00) |
|  |  |  |  |  |  |  |  |  |  |

Model 0 - unadjusted

Model 1 - adjusted for: sex, age, and ethnicity

Model 2 - model 1 also adjusted for: income, highest qualification, physical activity, sedentary behavior, total energy intake, body mass index, smoking status, and alcohol intake

Model 3 - model 2 also adjusted for: total sugar intake and total fat intake (total sugar intake was not included in the analysis of sugar-sweetened beverages)

Model 4 - model 3 also adjusted for: fresh fruit intake, vegetables intake, total fibre intake, red meat intake and processed meat intake

N number; HR hazard ratio; CI confidence interval

Supplementary table 2b. Cox proportional hazard model of the association between total sugar consumption and all-cause mortality - Landmark

|  | Second quintile | Third quintile | Forth quintile | Highest quintile |
| --- | --- | --- | --- | --- |
|  | 82g-105g | 106g-126g | 127g-154g | 155g-1,051g |
|  |  |  |  |  |
|  | n=39,655 | n=39,663 | n=39,650 | n=39,657 |
|  |  |  |  |  |
|  | HR (95% CI) | HR (95% CI) | HR (95% CI) | HR (95% CI) |
|  |  |  |  |  |
|  |  |  |  |  |
| 0 | 0.99 (0.88-1.11) | 0.94 (0.84-1.06) | 0.98 (0.87-1.10) | 1.15 (1.02-1.28) |
| 1 | 0.92 (0.82-1.03) | 0.85 (0.75-0.95) | 0.85 (0.75-0.95) | 0.96 (0.85-1.07) |
| 2 | 1.05 (0.91-1.20) | 1.00 (0.86-1.15) | 1.04 (0.90-1.21) | 1.19 (1.01-1.40) |
| 3 | 1.07 (0.93-1.23) | 1.03 (0.89-1.20) | 1.09 (0.93-1.28) | 1.28 (1.06-1.55) |
| 4 | 1.08 (0.94-1.24) | 1.05 (0.90-1.22) | 1.11 (0.94-1.31) | 1.31 (1.07-1.60) |

Model 0 - unadjusted

Model 1 - adjusted for: sex, age, and ethnicity

Model 2 - model 1 also adjusted for: income, highest qualification, physical activity, sedentary behavior, total energy intake, body mass index, smoking status, and alcohol intake

Model 3 - model 2 also adjusted for: total sugar intake and total fat intake

Model 4 - model 3 also adjusted for: fresh fruit intake, vegetables intake, total fibre intake, red meat intake and processed meat intake

N number; HR hazard ratio; CI confidence interval
