# Supplementary material for: The associations of sugar-sweetened, artificially sweetened and naturally sweet juices with all-cause mortality in 198,285 UK Biobank participants: a prospective cohort study
Source: BMC Med. 2020 Apr 24;18:97. doi: 10.1186/s12916-020-01554-5 (PMC7181499; doi:10.1186/s12916-020-01554-5)
Supplement: Supplementary file 3 — Additional file 3:Supplementary Table 3a. Cox proportional hazards models of the associations between categories of beverage intake and all-cause mortality -first intake. Supplementary Table 3b. Cox proportional hazard model of the association between total sugar consumption and all-cause mortality (first intake). [file 12916_2020_1554_MOESM3_ESM.docx]

Supplementary Table 3a. Cox proportional hazards models of the associations between categories of beverage intake and all-cause mortality -first intake

|  |  | |  |  | |  |  | |  |
| --- | --- | --- | --- | --- | --- | --- | --- | --- | --- |
|  | Sugar-sweetened beverages | |  | Artificially-sweetened beverages | |  | Fruit or vegetable juice | |  |
| Model | 1/day | >1-2/day | >2/day | 1/day | >1-2/day | >2/day | 1/day | >1-2/day | >2/day |
|  | n=27,841 | n=8,485 | n=4,541 | n=16,980 | n=6,183 | n=3,805 | n=70,871 | n=9,809 | n=2,378 |
|  |  |  |  |  |  |  |  |  |  |
|  | HR (95% CI) | HR (95% CI) | HR (95% CI) | HR (95% CI) | HR (95% CI) | HR (95% CI) | HR (95% CI) | HR (95% CI) | HR (95% CI) |
|  |  |  |  |  |  |  |  |  |  |
|  |  |  |  |  |  |  |  |  |  |
| 0 | 1.16 (1.05-1.28) | 1.11 (0.94-1.32) | 1.40 (1.15-1.72) | 0.91 (0.80-1.04) | 1.13 (0.93-1.36) | 1.14 (0.90-1.44) | 0.97 (0.90-1.05) | 0.85 (0.71-1.06) | 0.78 (0.54-1.11) |
| 1 | 1.19 (1.08 -1.31) | 1.26 (1.06-1.49) | 1.78 (1.44-2.17) | 1.07 (0.94-1.22) | 1.41 (1.17-1.71) | 1.58 (1.24-2.01) | 0.88 (0.81-0.94) | 0.82 (0.67-0.98) | 0.78 (0.54-1.11) |
| 2 | 1.15 (1.02-1.29) | 1.40 (1.16-1.70) | 1.50 (1.16-1.93) | 0.97 (0.83-1.14) | 1.33 (1.07-1.66) | 1.33 (1.00-1.78) | 0.95 (0.87-1.03) | 0.90 (0.74-1.10) | 0.67 (0.42-1.05) |
| 3 | 1.15 (1.02-1.29) | 1.41 (1.17-1.70) | 1.51 (1.17-1.96) | 0.98 (0.83-1.14) | 1.34 (1.07-1.67) | 1.34 (1.00-1.84) | 0.93 (0.85-1.02) | 0.87 (0.71-1.06) | 0.63 (0.40-0.99) |
| 4 | 1.15 (1.02-1.29) | 1.39 (1.15-1.67) | 1.49 (1.15-1.93) | 0.98 (0.83-1.15) | 1.34 (1.07-1.67) | 1.33 (1.00-1.77) | 0.93 (0.85-1.01) | 0.86 (0.70-1.05) | 0.61 (0.39-0.97) |
|  |  |  |  |  |  |  |  |  |  |

Model 0 - unadjusted

Model 1 - adjusted for: sex, age, and ethnicity

Model 2 - model 1 also adjusted for: income, highest qualification, physical activity, sedentary behavior, total energy intake, body mass index, smoking status, and alcohol intake

Model 3 - model 2 also adjusted for: total sugar intake and total fat intake (total sugar intake was not included in the analysis of sugar-sweetened beverages)

Model 4 - model 3 also adjusted for: fresh fruit intake, vegetables intake, total fibre intake, red meat intake and processed meat intake

N number; HR hazard ratio; CI confidence interval

Supplementary Table 3b. Cox proportional hazard model of the association between total sugar consumption and all-cause mortality (first intake)

|  | Second quintile | Third quintile | Forth quintile | Highest quintile |
| --- | --- | --- | --- | --- |
|  | 79g-103g | 104g-127g | 128g-159g | 160g-1,342g |
|  |  |  |  |  |
|  | n=39,662 | n=39,673 | n=39,638 | n=39,654 |
|  |  |  |  |  |
| Model | HR (95% CI) | HR (95% CI) | HR (95% CI) | HR (95% CI) |
|  |  |  |  |  |
|  |  |  |  |  |
| 0 | 1.02 (0.91-1.14) | 0.98 (0.88-1.10) | 1.01 (0.90-1.13) | 1.11 (1.00-1.24) |
| 1 | 0.96 (0.86-1.08) | 0.88 (0.79-0.99) | 0.89 (0.80-1.00) | 0.95 (0.85-1.06) |
| 2 | 1.11 (0.97-1.27) | 1.00 (0.87-1.15) | 1.08 (0.94-1.25) | 1.11 (0.95-1.30) |
| 3 | 1.12 (0.98-1.28) | 1.02 (0.88-1.18) | 1.11 (0.96-1.29) | 1.16 (0.97-1.39) |
| 4 | 1.13 (0.99-1.30) | 1.04 (0.90-1.20) | 1.14 (0.97-1.33) | 1.20 (0.99-1.45) |

Model 0 - unadjusted

Model 1 - adjusted for: sex, age, and ethnicity

Model 2 - model 1 also adjusted for: income, highest qualification, physical activity, sedentary behavior, total energy intake, body mass index, smoking status, and alcohol intake

Model 3 - model 2 also adjusted for: total sugar intake and total fat intake (total sugar intake was not included in the analysis of sugar-sweetened beverages)

Model 4 - model 3 also adjusted for: fresh fruit intake, vegetables intake, total fibre intake, red meat intake and processed meat intake

N number; HR hazard ratio; CI confidence interval
