# Supplementary material for: The associations of sugar-sweetened, artificially sweetened and naturally sweet juices with all-cause mortality in 198,285 UK Biobank participants: a prospective cohort study
Source: BMC Med. 2020 Apr 24;18:97. doi: 10.1186/s12916-020-01554-5 (PMC7181499; doi:10.1186/s12916-020-01554-5)
Supplement: Supplementary file 5 — Additional file 5:Supplementary Table 5. Cox proportional hazards models of the associations between categories of beverage intake and all-cause mortality –replacing Model 3 and model 4 with a diet quality score (score of processed meat, red meat, fruit, vegetable, fat and sugar intake). [file 12916_2020_1554_MOESM5_ESM.docx]

Supplementary table 5. Cox proportional hazards models of the associations between categories of beverage intake and all-cause mortality –replacing Model 3 and model 4 with a diet quality score (score of processed meat, red meat, fruit, vegetable and fat intake)

|  |  | |  |  | |  |  | |  |
| --- | --- | --- | --- | --- | --- | --- | --- | --- | --- |
|  | Sugar-sweetened beverages | |  | Artificially-sweetened beverages | |  | Fruit or vegetable juice | |  |
| Model | 1/day | >1-2/day | >2/day | 1/day | >1-2/day | >2/day | 1/day | >1-2/day | >2/day |
|  | n=51,842 | n=9,415 | n=3,770 | n=27,079 | n=8,680 | n=5,032 | n=89,206 | n=12,492 | n=2,019 |
|  |  |  |  |  |  |  |  |  |  |
|  | HR (95% CI) | HR (95% CI) | HR (95% CI) | HR (95% CI) | HR (95% CI) | HR (95% CI) | HR (95% CI) | HR (95% CI) | HR (95% CI) |
|  |  |  |  |  |  |  |  |  |  |
|  |  |  |  |  |  |  |  |  |  |
| 0 | 1.03 (0.95-1.12) | 1.10 (0.94-1.30) | 1.71 (1.40-2.09) | 0.84 (0.75-0.93) | 1.06 (0.90-1.26) | 1.24 (1.01-1.52) | 0.89 (0.83-0.95) | 0.84 (0.72-0.98) | 0.73 (0.49-1.08) |
| 1 | 1.07 (0.98-1.16) | 1.28 (1.09-1.51) | 2.13 (1.74-2.62) | 0.98 (0.88-1.09) | 1.35 (1.14-1.60) | 1.73 (1.41-2.12) | 0.82 (0.77-0.89) | 0.80 (0.67-0.94) | 0.76 (0.49-1.08) |
| 2 | 1.06 (0.97-1.17) | 1.35 (1.12-1.62) | 1.86 (1.44-2.40) | 0.92 (0.81-1.05) | 1.13 (0.91-1.39) | 1.44 (1.12-1.84) | 0.91 (0.83-0.99) | 0.89 (0.74-1.06) | 0.64 (0.39-1.05) |
| 3 | 1.06 (0.97-1.17) | 1.35 (1.12-1.62) | 1.86 (1.45-2.40) | 0.92 (0.81-1.05) | 1.13 (0.91-1.39) | 1.44 (1.12-1.84) | 0.91 (0.83-0.99) | 0.89 (0.75-1.07) | 0.65 (0.39-1.06) |
|  |  |  |  |  |  |  |  |  |  |

Model 0 - unadjusted

Model 1 - adjusted for: sex, age, and ethnicity

Model 2 - model 1 also adjusted for: income, highest qualification, physical activity, sedentary behavior, total energy intake, body mass index, smoking status, and alcohol intake

Model 3 - model 2 also adjusted for a diet quality score

N number; HR hazard ratio; CI confidence interval
