# Supplementary material for: The associations of sugar-sweetened, artificially sweetened and naturally sweet juices with all-cause mortality in 198,285 UK Biobank participants: a prospective cohort study
Source: BMC Med. 2020 Apr 24;18:97. doi: 10.1186/s12916-020-01554-5 (PMC7181499; doi:10.1186/s12916-020-01554-5)
Supplement: Supplementary file 6 — Additional file 6:Supplementary Table 6.a. Cox proportional hazards model4 of the associations between categories of beverage intake and all-cause mortality with or without adjustment for total energy. Supplementary Table 6.b. Cox proportional hazards model4 of the associations between categories of beverage intake and all-cause mortality with or without adjustment for BMI. [file 12916_2020_1554_MOESM6_ESM.docx]

Supplementary table 6.a Cox proportional hazards model4 of the associations between categories of beverage intake and all-cause mortality with or without adjustment for total energy

|  |  | |  |  | |  |  | |  |
| --- | --- | --- | --- | --- | --- | --- | --- | --- | --- |
|  | Sugar-sweetened beverages | |  | Artificially-sweetened beverages | |  | Fruit or vegetable juice | |  |
| Model | 1/day | >1-2/day | >2/day | 1/day | >1-2/day | >2/day | 1/day | >1-2/day | >2/day |
|  | n=51,842 | n=9,415 | n=3,770 | n=27,079 | n=8,680 | n=5,032 | n=89,206 | n=12,492 | n=2,019 |
|  |  |  |  |  |  |  |  |  |  |
|  | HR (95% CI) | HR (95% CI) | HR (95% CI) | HR (95% CI) | HR (95% CI) | HR (95% CI) | HR (95% CI) | HR (95% CI) | HR (95% CI) |
|  |  |  |  |  |  |  |  |  |  |
|  |  |  |  |  |  |  |  |  |  |
| 4 | 1.06 (0.96-1.16) | 1.33 (1.10-1.60) | 1.84 (1.42-2.37) | 0.92 (0.81-1.05) | 1.13 (0.91-1.39) | 1.44 (1.12-1.84) | 0.89 (0.81-0.97) | 0.83 (0.69-1.00) | 0.57 (0.35-0.93) |
| 4-total E | 1.05 (0.96-1.16) | 1.32 (1.10-1.59) | 1.81 (1.40-2.33) | 0.92 (0.81-1.05) | 1.13 (0.91-1.39) | 1.44 (1.12-1.84) | 0.89 (0.81-0.97) | 0.83 (0.69-1.00) | 0.56 (0.34-0.93) |
|  |  |  |  |  |  |  |  |  |  |

Model 4 - adjusted for: sex, age, and ethnicity, income, highest qualification, physical activity, sedentary behavior, total energy intake, body mass index, smoking status, and alcohol intake, total sugar intake and total fat intake, fresh fruit intake, vegetables intake, total fibre intake, red meat intake and processed meat intake (total sugar variable was not included in the analysis of sugar-sweetened beverages)

Model4-total E: adjusted as model 4 without total energy intake

N number; HR hazard ratio; CI confidence interval

Supplementary table 6.b Cox proportional hazards model4 of the associations between categories of beverage intake and all-cause mortality with or without adjustment for BMI

|  |  | |  |  | |  |  | |  |
| --- | --- | --- | --- | --- | --- | --- | --- | --- | --- |
|  | Sugar-sweetened beverages | |  | Artificially-sweetened beverages | |  | Fruit or vegetable juice | |  |
| Model | 1/day | >1-2/day | >2/day | 1/day | >1-2/day | >2/day | 1/day | >1-2/day | >2/day |
|  | n=51,842 | n=9,415 | n=3,770 | n=27,079 | n=8,680 | n=5,032 | n=89,206 | n=12,492 | n=2,019 |
|  |  |  |  |  |  |  |  |  |  |
|  | HR (95% CI) | HR (95% CI) | HR (95% CI) | HR (95% CI) | HR (95% CI) | HR (95% CI) | HR (95% CI) | HR (95% CI) | HR (95% CI) |
|  |  |  |  |  |  |  |  |  |  |
|  |  |  |  |  |  |  |  |  |  |
| 4 | 1.06 (0.96-1.16) | 1.33 (1.10-1.60) | 1.84 (1.42-2.37) | 0.92 (0.81-1.05) | 1.13 (0.91-1.39) | 1.44 (1.12-1.84) | 0.89 (0.81-0.97) | 0.83 (0.69-1.00) | 0.57 (0.35-0.93) |
| 4 excl BMI | 1.07 (0.97-1.18) | 1.34 (1.11-1.61) | 1.89 (1.47-2.43) | 0.95 (0.84-1.08) | 1.21 (0.98-1.48) | 1.56 (1.22-1.98) | 0.87 (0.80-0.95) | 0.83 (0.69-0.99) | 0.60 (0.37-0.97) |
|  |  |  |  |  |  |  |  |  |  |

Model 4 - adjusted for: sex, age, and ethnicity, income, highest qualification, physical activity, sedentary behavior, total energy intake, body mass index, smoking status, and alcohol intake, total sugar intake and total fat intake, fresh fruit intake, vegetables intake, total fibre intake, red meat intake and processed meat intake (total sugar variable was not included in the analysis of sugar-sweetened beverages)

Model4-excl BMI: adjusted as model 4 without BMI

N number; HR hazard ratio; CI confidence interval
