# Supplementary material for: The associations of sugar-sweetened, artificially sweetened and naturally sweet juices with all-cause mortality in 198,285 UK Biobank participants: a prospective cohort study
Source: BMC Med. 2020 Apr 24;18:97. doi: 10.1186/s12916-020-01554-5 (PMC7181499; doi:10.1186/s12916-020-01554-5)
Supplement: Supplementary file 7 — Additional file 7:Supplementary Table 7. Cox proportional hazards -Model 4- of the associations between categories of beverage intake and all-cause mortality stratified by BMI group. [file 12916_2020_1554_MOESM7_ESM.docx]

Supplementary table 7. Cox proportional hazards of the associations between categories of beverage intake and all-cause mortality stratified by BMI group – Model 4

|  |  | |  |  | |  |  | |  |
| --- | --- | --- | --- | --- | --- | --- | --- | --- | --- |
|  | Sugar-sweetened beverages | |  | Artificially-sweetened beverages | |  | Fruit or vegetable juice | |  |
| BMI group | 1/day | >1-2/day | >2/day | 1/day | >1-2/day | >2/day | 1/day | >1-2/day | >2/day |
|  |  |  |  |  |  |  |  |  |  |
|  |  |  |  |  |  |  |  |  |  |
|  | HR (95% CI) | HR (95% CI) | HR (95% CI) | HR (95% CI) | HR (95% CI) | HR (95% CI) | HR (95% CI) | HR (95% CI) | HR (95% CI) |
|  |  |  |  |  |  |  |  |  |  |
|  |  |  |  |  |  |  |  |  |  |
| Normal | 0.88 (0.73-1.06) | 0.97 (0.64-1.47) | 1.50 (0.84-2.67) | 0.92 (0.70-1.21) | 1.14 (0.69-1.87) | 1.45 (0.77 -2.71) | 0.92 (0.78-1.07) | 0.94 (0.69-1.29) | 0.41 (0.13-1.30) |
| Overweight | 1.14 (1.00-1.32) | 1.36 (1.02-1.82) | 1.97 (1.33-2.92) | 0.88 (0.72-1.07) | 1.11 (0.80-1.55) | 1.56 (1.04-2.32) | 0.92 (0.80-1.05) | 0.82 (0.62-1.08) | 0.65 (0.32-1.33) |
| Obese | 1.13 (0.93-1.36) | 1.66 (1.21-2.28) | 1.99 (1.30-3.03) | 0.99 (0.79-1.24) | 1.16 (0.84-1.61) | 1.38 (0.96-1.99) | 0.81 (0.68-0.97) | 0.76 (0.52-1.10) | 0.60 (0.25-1.48) |

Model 4 - adjusted for: sex, age, and ethnicity, income, highest qualification, physical activity, sedentary behavior, total energy intake, body mass index, smoking status, and alcohol intake, total sugar intake and total fat intake, fresh fruit intake, vegetables intake, total fibre intake, red meat intake and processed meat intake. Total sugar intake is not included in the analysis of sugar-sweetened beverages.

BMI group: Normal n=72,364, dead n=901; Overweight n=82,532, dead n=1,325; Obese n=39,878, dead n=824; there were not enough cases in the Underweight group.

N number; HR hazard ratio; CI confidence interval
