# Supplementary material for: The associations of sugar-sweetened, artificially sweetened and naturally sweet juices with all-cause mortality in 198,285 UK Biobank participants: a prospective cohort study
Source: BMC Med. 2020 Apr 24;18:97. doi: 10.1186/s12916-020-01554-5 (PMC7181499; doi:10.1186/s12916-020-01554-5)
Supplement: Supplementary file 8 — Additional file 8:Supplementary Table 8. Cox proportional hazards models of the associations between categories of beverage intake and all-cause mortality with added Model 5 –adjusted for prevalent disease at baseline (cancer N = 15,121), other conditions (N = 6333), Model 6 -the same conditions excluded instead. [file 12916_2020_1554_MOESM8_ESM.docx]

Supplementary Table 8. Cox proportional hazards models of the associations between categories of beverage intake and all-cause mortality with added Model 5 –adjusted for prevalent disease at baseline (cancer N=15,121), other conditions* (N=6,333), Model 6 - participants with the same conditions excluded rather than adjusted

|  |  | |  |  | |  |  | |  |
| --- | --- | --- | --- | --- | --- | --- | --- | --- | --- |
|  | Sugar-sweetened beverages | |  | Artificially-sweetened beverages | |  | Fruit or vegetable juice | |  |
| Model | 1/day | >1-2/day | >2/day | 1/day | >1-2/day | >2/day | 1/day | >1-2/day | >2/day |
|  | n=51,842 | n=9,415 | n=3,770 | n=27,079 | n=8,680 | n=5,032 | n=89,206 | n=12,492 | n=2,019 |
|  |  |  |  |  |  |  |  |  |  |
|  | HR (95% CI) | HR (95% CI) | HR (95% CI) | HR (95% CI) | HR (95% CI) | HR (95% CI) | HR (95% CI) | HR (95% CI) | HR (95% CI) |
|  |  |  |  |  |  |  |  |  |  |
|  |  |  |  |  |  |  |  |  |  |
| 0 | 1.03 (0.95-1.12) | 1.10 (0.94-1.30) | 1.71 (1.40-2.09) | 0.84 (0.75-0.93) | 1.06 (0.90-1.26) | 1.24 (1.01-1.52) | 0.89 (0.83-0.95) | 0.84 (0.72-0.98) | 0.73 (0.49-1.08) |
| 1 | 1.07 (0.98-1.16) | 1.28 (1.09-1.51) | 2.13 (1.74-2.62) | 0.98 (0.88-1.09) | 1.35 (1.14-1.60) | 1.73 (1.41-2.12) | 0.82 (0.77-0.89) | 0.80 (0.67-0.94) | 0.76 (0.49-1.08) |
| 2 | 1.06 (0.97-1.17) | 1.35 (1.12-1.62) | 1.86 (1.44-2.40) | 0.92 (0.81-1.05) | 1.13 (0.91-1.39) | 1.44 (1.12-1.84) | 0.91 (0.83-0.99) | 0.89 (0.74-1.06) | 0.64 (0.39-1.05) |
| 3 | 1.06 (0.96-1.16) | 1.31 (1.09-1.59) | 1.78 (1.37-2.31) | 0.92 (0.81-1.05) | 1.13 (0.91-1.39) | 1.44 (1.12-1.84) | 0.89 (0.82-0.97) | 0.84 (0.70-1.01) | 0.58 (0.35-0.96) |
| 4 | 1.06 (0.96-1.16) | 1.33 (1.10-1.60) | 1.84 (1.42-2.37) | 0.92 (0.81-1.05) | 1.13 (0.91-1.39) | 1.44 (1.12-1.84) | 0.89 (0.81-0.97) | 0.83 (0.69-1.00) | 0.57 (0.35-0.93) |
| 5 | 1.05 (0.95-1.17) | 1.34 (1.09-1.64) | 1.81 (1.37-2.39) | 0.99 (0.86-1.14) | 1.14 (0.91-1.44) | 1.43 (1.10-1.86) | 0.88 (0.80-0.97) | 0.82 (0.67-1.00) | 0.42 (0.23-0.80) |
| 6 | 1.04 (0.93-1.17) | 1.18 (0.94-1.51) | 1.84 (1.35-2.52) | 1.00 (0.86-1.17) | 1.06 (0.81-1.38) | 1.57 (1.17-2.10) | 0.88 (0.80-0.98) | 0.80 (0.64-1.00) | 0.65 (0.36-1.16) |

*COPD, emphysema, pulmonary fibrosis, Heart attack, Heart failure, Rheumatoid arthritis

Model 0 - unadjusted

Model 1 - adjusted for: sex, age, and ethnicity

Model 2 - model 1 also adjusted for: income, highest qualification, physical activity, sedentary behavior, total energy intake, body mass index, smoking status, and alcohol intake

Model 3 - model 2 also adjusted for: total sugar intake and total fat intake (total sugar intake not included in the analysis of sugar-sweetened beverages)

Model 4 - model 3 also adjusted for: fresh fruit intake, vegetables intake, total fibre intake, red meat intake and processed meat intake

Model 5 –adjusted for prevalent disease at baseline -cancer N=15,121), other conditions* (N=6,333)

Model 6 -participants with the same conditions excluded rather than adjusted

N number; HR hazard ratio; CI confidence interval
