# Supplementary material for: The associations of sugar-sweetened, artificially sweetened and naturally sweet juices with all-cause mortality in 198,285 UK Biobank participants: a prospective cohort study
Source: BMC Med. 2020 Apr 24;18:97. doi: 10.1186/s12916-020-01554-5 (PMC7181499; doi:10.1186/s12916-020-01554-5)
Supplement: Supplementary file 10 — Additional file 10:Supplementary Table 10. Cox proportional hazard model of the association between artificially-sweetened drinks and all-cause mortality, excluding those with weight loss one year prior baseline recruitment or missing information (N = 31,642). [file 12916_2020_1554_MOESM10_ESM.docx]

Supplementary table 10. Cox proportional hazard model of the association between artificially-sweetened drinks and all-cause mortality, excluding those with weight loss one year prior baseline recruitment or missing information (N=31,642)

|  | Artificially-sweetened drinks/day | | |
| --- | --- | --- | --- |
|  | <=1 | >1-2 | >2 |
|  |  |  |  |
|  | n=22,089 | n=6,783 | n=3,742 |
| Model |  |  |  |
|  | HR (95% CI) | HR (95% CI) | HR (95% CI) |
|  |  |  |  |
|  |  |  |  |
| 0 | 0.82 (0.72-0.92) | 1.05 (0.90-1.26) | 1.18 (0.92-1.51) |
| 1 | 0.95 (0.84-1.07) | 1.33 (1.09-1.62) | 1.66 (1.30-2.13) |
| 2 | 0.85 (0.74-0.99) | 1.22 (0.97-1.54) | 1.33 (0.99-1.80) |
| 3 | 0.86 (0.74-0.99) | 1.22 (0.97-1.54) | 1.34 (0.99-1.81) |
| 4 | 0.86 (0.74-0.99) | 1.22 (0.96-1.54) | 1.34 (0.99-1.81) |

Model 0 - unadjusted

Model 1 - adjusted for: sex, age, and ethnicity

Model 2 - model 1 also adjusted for: income, highest qualification, physical activity, sedentary behavior, total energy intake, body mass index, smoking status, and alcohol intake

Model 3 - model 2 also adjusted for: total sugar intake and total fat intake

Model 4 - model 3 also adjusted for: fresh fruit intake, vegetables intake, total fibre intake, red meat intake and processed meat intake

N number; HR hazard ratio; CI confidence interval
