# Supplementary material for: The associations of sugar-sweetened, artificially sweetened and naturally sweet juices with all-cause mortality in 198,285 UK Biobank participants: a prospective cohort study
Source: BMC Med. 2020 Apr 24;18:97. doi: 10.1186/s12916-020-01554-5 (PMC7181499; doi:10.1186/s12916-020-01554-5)
Supplement: Supplementary file 11 — Additional file 11:Supplementary Table 11. Cox proportional hazards -Model 4- of the associations between categories of beverage intake and all-cause mortality stratified by all covariates. [file 12916_2020_1554_MOESM11_ESM.docx]

Supplementary table 11. Cox proportional hazards of the associations between categories of beverage intake and all-cause mortality stratified by all covariates – Model 4

|  |  |  |  |  |  |  |  |  |  |
| --- | --- | --- | --- | --- | --- | --- | --- | --- | --- |
|  | Sugar-sweetened beverages |  |  | Artificially-sweetened beverages |  |  | Fruit or vegetable juice |  |  |
|  | 1/day | >1-2/day | >2/day | 1/day | >1-2/day | >2/day | 1/day | >1-2/day | >2/day |
|  |  |  |  |  |  |  |  |  |  |
|  |  |  |  |  |  |  |  |  |  |
|  | HR (95% CI) | HR (95% CI) | HR (95% CI) | HR (95% CI) | HR (95% CI) | HR (95% CI) | HR (95% CI) | HR (95% CI) | HR (95% CI) |
|  |  |  |  |  |  |  |  |  |  |
| **Sex** |  |  |  |  |  |  |  |  |  |
| Men | 1.02 (0.90-1.16) | 1.30 (1.02-1.64) | 1.80 (1.32-2.46) | 0.98 (0.83-1.16) | 1.11 (0.83 -1.50) | 1.91 (1.42-2.58) | 0.93 (0.83-1.04) | 0.89 (0.71-1.12) | 0.71 (0.40-1.26) |
| Women | 1.12 (0.96-1.30) | 1.41 (1.03-1.92) | 1.90 (1.20-3.00) | 0.84 (0.69 - 1.03) | 1.12 (0.83-1.52) | 0.92 (0.59-1.42) | 0.87 (0.76-1.00) | 0.86 (0.64-1.16) | 0.48 (0.18-1.29) |
| **Age** |  |  |  |  |  |  |  |  |  |
| <55 | 1.05 (0.84-1.30) | 0.88 (0.57-1.36) | 1.49 (0.91-2.44) | 0.82 (0.62-1.09) | 0.81 (0.52 - 1.26) | 0.91 (0.55 - 1.52) | 1.07 (0.85-1.31) | 0.90 (0.59-1.39) | 0.86 (0.32-2.34) |
| >55 | 1.04 (0.94-1.16) | 1.39 (1.13-1.71) | 1.80 (1.33-2.42) | 0.89 (0.77 - 1.03) | 1.13 (0.89 - 1.44) | 1.45 (1.09 - 1.93) | 0.89 (0.81-0.98) | 0.89 (0.73-1.08) | 0.59 (0.33-1.05) |
| **Ethnicity** |  |  |  |  |  |  |  |  |  |
| White | 1.07 (0.97-1.75) | 1.33 (1.10-1.61) | 1.85 (1.43-2.40) | 0.93 (0.82 - 1.06) | 1.14 (0.92 -1.41) | 1.44 (1.12 - 1.84) | 0.91 (0.83-0.99) | 0.86 (0.71-1.03) | 0.65 (0.40-1.08) |
| **Income** |  |  |  |  |  |  |  |  |  |
| <18,000 | 1.03 (0.84-1.27) | 1.12 (0.74-1.69) | 2.20 (1.40-3.44) | 0.87 (0.65-1.16) | 1.29 (0.84-1.97) | 1.29 (0.79-2.12) | 0.75 (0.62 -0.90) | 0.82 (0.55-1.23) | 0.69 (0.25-1.87) |
| 18,000-30,999 | 0.97 (0.82-1.16) | 1.17 (0.81-1.68) | 2.41 (1.58-2.37) | 1.13 (0.90-1.41) | 1.20 (0.81 - 1.77) | 1.47 (0.91 -2.37) | 0.84 (0.72-0.99) | 1.01 (0.73-0.99) | 0.40 (0.13-1.26) |
| 31,000-50,999 | 1.01 (0.83-1.21) | 1.54 (1.09-2.17) | 1.32 (0.73-2.37) | 0.78 (0.59 - 1.00) | 1.15 (0.78 - 1.71) | 1.63 (1.03 - 2.57) | 1.03 (0.87-1.21) | 0.81 (0.57-1.17) | 1.04 (0.49-2.23) |
| 52,000-100,000 | 1.20 (0.95-1.51) | 1.74 (1.13-2.67) | 1.24 (0.55-2.83) | 1.00 (0.74 - 1.36) | 1.06 (0.63 - 1.80) | 1.51 (0.82 - 2.78) | 1.01 (0.82-1.26) | 0.91 (0.60-1.38) | 0.23 (0.03-1.66) |
| >100,000 | 1.75 (1.15-2.68) | 0.83 (0.26-2.70) | N/A | 0.58 (0.29-1.16) | N/A | N/A | 1.09 (0.70-1.68) | 0.67 (0.29-1.55) | 0.67 (0.09-4.98) |
| **Education** |  |  |  |  |  |  |  |  |  |
| higher degree | 1.20 (1.05-1.38) | 1.52 (1.14-2.03) | 2.02 (1.31-3.11) | 0.92 (0.76-1.12) | 0.71 (0.46-1.07) | 1.98 (1.40-2.77) | 0.93 (0.82-1.06) | 0.70 (0.53-0.91) | 0.63 (0.32-1.22) |
| any degree | 0.96 (0.83-1.12) | 1.24 (0.93-1.66) | 1.60 (1.09-2.35) | 0.82 (0.66-1.01) | 1.50 (1.14 -1.98) | 1.10 (0.73-1.67) | 0.86 (0.75-0.98) | 0.99 (0.74-1.31) | 0.57 (0.23-1.38) |
| professional | 0.77 (0.54-1.11) | 0.83 (0.40-1.72) | 1.37 (0.59-3.19) | 1.24 (0.83-1.88) | 1.44 (0.75 -2.76) | 0.76 (0.4-2.40) | 0.93 (0.68-1.27) | 1.42 (0.78-2.57) | N/A |
| other | 1.10 (0.74-1.64) | 1.54 (0.76-3.11) | 3.32 (1.50-7.36) | 1.21 (0.75-1.94) | 0.64 (0.20-2.02) | 1.74 (0.63-4.79) | 0.95 (0.67-1.36) | 1.45 (0.75-2.79) | 1.58 (0.37-6.66) |
| **Physical activity** |  |  |  |  |  |  |  |  |  |
| low | 1.06 (0.91-1.24) | 1.24 (0.91-1.69) | 2.15 (1.50-3.07) | 1.00 (0.81-1.22) | 1.11 (0.79 -1.54) | 1.32 (0.89-1.94) | 0.79 (0.69-0.90) | 0.94 (0.71-1.23) | 0.66 (0.33-1.35) |
| med | 1.08 (0.92-1.27) | 1.44 (1.04-2.02) | 1.27 (0.69-2.33) | 0.89 (0.71 - 1.11) | 1.18 (0.81 -1.71) | 1.37 (0.87 - 2.14) | 0.97 (0.61-1.16) | 0.84 (0.61-1.16) | 1.00 (0.47-2.13) |
| high | 1.02 (0.85-1.22) | 1.32 (0.94-1.85) | 1.83 (1.14-2.93) | 0.85 (0.66-1.09) | 1.06 (0.71-1.59) | 1.71 (1.09-2.70) | 1.01 (0.86-1.20) | 0.83 (0.58-1.17) | 0.14 (0.02-1.03) |
| **Sedentary behaviour** |  |  |  |  |  |  |  |  |  |
| low | 0.93 (0.77-1.11) | 1.27 (0.87-1.85) | 1.47 (0.82-2.64) | 0.83 (0.63-1.08) | 0.96 (0.61-1.53) | 1.39 (0.80-2.42) | 1.11 (0.95-1.31) | 0.75 (0.52-1.07) | 0.81 (0.36-1.83) |
| med | 1.13 (0.96-1.33) | 1.42 (1.03-1.96) | 2.05 (1.32-3.17) | 1.00 (0.81-1.25) | 1.52 (1.10-2.10) | 1.04 (0.62-1.74) | 0.82 (0.71-0.96) | 0.96 (0.71-1.29) | 0.38 (0.12-1.18) |
| high | 1.10 (0.95-1.29) | 1.33 (0.99-1.78) | 1.94 (1.33-2.84) | 0.90 (0.74-1.11) | 0.94 (0.66-1.33) | 1.75 (1.26-2.43) | 0.83 (0.72-0.96) | 0.91 (0.68-1.22) | 0.69 (0.32-1.46) |
| **Smoking** |  |  |  |  |  |  |  |  |  |
| never | 1.14 (0.99-1.32) | 1.48 (1.12-1.96) | 1.77 (1.18-2.67) | 0.95 (0.78-1.15) | 0.94 (0.65-1.34) | 1.64 (1.13-2.37) | 0.92 (0.81-1.05) | 0.96 (0.75-1.25) | 0.68 (0.34-1.38) |
| past | 0.94 (0.81-1.10) | 1.34 (1.01-1.78) | 2.07 (1.42-3.01) | 0.90 (0.74-1.09) | 1.24 (0.92-1.67) | 1.56 (1.10-2.23) | 0.89 (0.78-1.01) | 0.74 (0.55-0.99) | 0.47 (0.20-1.15) |
| current | 1.17 (0.90-1.52) | 0.91 (0.51-1.60) | 1.49 (0.75-2.96) | 0.96 (0.67-1.39) | 1.33 (0.77-2.30) | 0.57 (0.21-1.53) | 0.89 (0.70-1.15) | 1.02 (0.63-1.65) | 1.06 (0.33-3.39) |
| **Total Energy** |  |  |  |  |  |  |  |  |  |
| low | 1.08 (0.90-1.29) | 1.51 (1.04-2.20) | 1.30 (0.67-2.52) | 0.88 (0.70-1.11) | 1.30 (0.93-1.82) | 1.20 (0.76-1.88) | 0.93 (0.80-1.08) | 1.07 (0.75-1.52) | 0.50 (0.12-2.00) |
| med | 1.17 (1.00-1.37) | 1.53 (1.11-2.11) | 2.19 (1.39-3.45) | 1.09 (0.88-1.34) | 1.16 (0.80-1.67) | 1.62 (1.05-2.50) | 0.92 (0.79-1.06) | 0.81 (0.59-1.11) | 0.31 (0.08-1.25) |
| high | 0.96 (0.81-1.12) | 1.12 (0.84-1.50) | 1.85 (1.30-2.62) | 0.79 (0.63-1.00) | 0.91 (0.61-1.36) | 1.48 (0.98-2.23) | 0.89 (0.76-1.03) | 0.86 (0.67 - 1.13) | 0.80 (0.45-1.43) |
| **BMI group** |  |  |  |  |  |  |  |  |  |
| Normal | 0.88 (0.73-1.05) | 0.97 (0.64-1.47) | 1.49 (0.83-2.66) | 0.92 (0.70-1.21) | 1.13 (0.69-1.87) | 1.45 (0.77 -2.71) | 0.92 (0.78-1.07) | 0.94 (0.68-1.29) | 0.41 (0.13-1.29) |
| Overweight | 1.15 1.00-1.33) | 1.39 (1.05-1.85) | 2.03 (1.37-3.00) | 0.88 (0.72-1.07) | 1.11 (0.80-1.55) | 1.56 (1.05-2.33) | 0.92 (0.81-1.08) | 0.66 (0.33-1.35) | 0.66 (0.33-1.35) |
| Obese | 1.13 (0.96-1.16) | 1.68 (1.22-1.30) | 2.01 (1.32-3.07) | 0.99 (0.79-1.24) | 1.16 (0.84-1.62) | 1.39 (0.96-2.00) | 0.81 (0.68-0.97) | 0.76 (0.52-1.10) | 0.61 (0.25-1.49) |
| **Alcohol** |  |  |  |  |  |  |  |  |  |
| low | 1.11 (0.94-1.31) | 1.22 (0.91-1.65) | 1.75 (1.21-2.51) | 0.86 (0.68-1.08) | 0.91 (0.65-1.29) | 1.65 (1.20-2.28) | 0.96 (0.83-1.12) | 1.16 (0.88-1.54) | 0.63 (0.31-1.27) |
| med | 1.03 (0.87-1.22) | 1.22 (0.85-1.77) | 2.37 (1.45-3.90) | 1.04 (0.84-1.29) | 1.44 (0.99-2.10) | 1.05 (0.56-1.98) | 1.03 (0.88-1.21) | 0.98 (0.69-1.33) | 0.37 (0.09-1.50) |
| high | 1.10 (0.93-1.29) | 1.55 (1.12-2.14) | 1.47 (0.86-2.53) | 0.92 (0.73-1.15) | 1.13 (0.77-1.66) | 1.24 (0.76-2.05) | 0.81 (0.70-0.94) | 0.61 (0.43-0.86) | 0.74 (0.33-1.67) |
| **Fat** |  |  |  |  |  |  |  |  |  |
| low | 1.18 (0.99-1.40) | 1.96 (1.43-2.71) | 2.46 (1.60-3.76) | 0.93 (0.74-1.17) | 1.18 (0.83-1.67) | 1.33 (0.87-2.05) | 0.95 (0.81-1.10) | 0.86 (0.61-1.20) | 0.63 (0.26-1.53) |
| med | 1.00 (0.85-1.18) | 1.37 (0.98-1.90) | 1.48 (0.88-2.49) | 0.93 (0.74-1.15) | 1.15 (0.79-1.67) | 1.53 (0.97-2.40) | 0.95 (0.81-1.10) | 1.01 (0.75-1.37) | 0.44 (0.14-1.38) |
| high | 1.02 (0.87-1.20) | 0.96 (0.70-1.33) | 1.70 (1.14-2.55) | 0.89 (0.71-1.12) | 1.03 (0.71-1.52) | 1.42 (0.95-2.14) | 0.85 (0.74-0.99) | 0.81 (0.60-1.09) | 0.78 (0.38-1.57) |
| **Vegetables** |  |  |  |  |  |  |  |  |  |
| low | 1.11 (0.95-1.29) | 1.26 (0.93-1.71) | 1.57 (1.04-2.37) | 0.92 (0.75-1.14) | 1.11 (0.79-1.56) | 1.76 (1.23-2.52) | 0.86 (0.75-0.99) | 0.94 (0.70-1.26) | 0.97 (0.50-1.89) |
| med | 1.02 (0.86-1.21) | 1.20 (0.83-1.73) | 2.11 (1.34-3.30) | 0.95 (0.75-1.20) | 1.09 (0.73-1.64) | 1.35 (0.83-2.21) | 0.84 (0.72-0.98) | 0.80 (0.58-1.11) | 0.28 (0.07-1.11) |
| high | 1.04 (0.87-1.24) | 1.54 (1.11-2.12) | 2.01 (1.25-3.25) | 0.89 (0.70-1.12) | 1.17 (0.81-1.68) | 1.13 (0.70-1.82) | 1.03 (0.88-1.20) | 0.89 (0.65-1.21) | 0.56 (0.23-1.36) |
| **Fruit** |  |  |  |  |  |  |  |  |  |
| low | 1.08 (0.93-1.25) | 1.11 (0.81-1.50) | 1.56 (1.03-2.35) | 0.96 (0.79-1.18) | 1.30 (0.94-1.79) | 1.48 (1.01-2.16) | 0.87 (0.76-1.00) | 1.01 (0.76-1.34) | 0.67 (0.31-1.55) |
| med | 1.09 (0.91-1.30) | 1.36 (0.95-1.96) | 1.82 (1.09-3.04) | 0.89 (0.69-1.15) | 1.32 (0.90-1.92) | 1.42 (0.85-2.39) | 0.84 (0.71-0.99) | 0.81 (0.57-1.14) | 0.42 (0.13-1.31) |
| high | 1.01 (0.85-1.20) | 1.66 (1.21-2.28) | 2.31 (1.50-3.55) | 0.90 (0.72-1.12) | 0.80 (0.52 - 1.21) | 1.43 (0.94-2.17) | 1.00 (0.86-1.17) | 0.80 (0.59-1.10) | 0.74 (0.35-1.58) |
| **Red meat** |  |  |  |  |  |  |  |  |  |
| low | 1.06 (0.93-1.22) | 1.09 (0.82-1.46) | 1.31 (0.87-1.96) | 0.90 (0.73-1.09) | 1.10 (0.82-1.49) | 1.56 (1.12-2.18) | 0.93 (0.83-1.05) | 0.75 (0.58-0.99) | 0.47 (0.22-0.99) |
| med | 1.20 (1.00-1.43) | 1.74 (1.22-2.47) | 2.34 (1.37-3.99) | 0.91 (0.72-1.14) | 1.01 (0.64-1.60) | 1.05 (0.57-1.93) | 0.92 (0.77-1.10) | 1.17 (0.85-1.61) | 1.17 (0.52-2.68) |
| high | 1.00 (0.82-1.22) | 1.47 (1.03-2.10) | 2.58 (1.69-3.97) | 1.03 (0.79-1.32) | 1.22 (0.83-1.81) | 1.44 (0.90-2.30) | 0.89 (0.74-1.05) | 0.84 (0.57-1.24) | 0.55 (0.17-1.71) |
| **Processed meat** |  |  |  |  |  |  |  |  |  |
| low | 1.14 (0.99-1.30) | 1.39 (1.07-1.82) | 1.93 (1.37-2.72) | 0.90 (0.74-1.09) | 0.85 (0.61-1.19) | 1.37 (0.97-1.93) | 0.90 (0.80-1.02) | 0.94 (0.73-1.20) | 0.80 (0.46-1.40) |
| med | 0.91 (0.71-1.18) | 0.88 (0.46-1.68) | 2.35 (1.13-4.88) | 0.92 (0.66-1.28) | 1.29 (0.72-2.33) | 1.81 (0.84-3.90) | 0.91 (0.71-1.16) | 0.79 (0.47-1.31) | 0.39 (0.06-2.84) |
| high | 1.06 (0.90-1.24) | 1.42 (1.06-1.90) | 1.56 (0.99-2.46) | 0.99 (0.80-1.21) | 1.50 (1.10-2.04) | 1.45 (0.96-2.17) | 0.92 (0.80-1.07) | 0.83 (0.61-1.12) | 0.28 (0.07-1.13) |
| **Fibre** |  |  |  |  |  |  |  |  |  |
| low | 1.05 (0.89-1.24) | 1.42 (1.05-1.93) | 1.53 (0.98-2.39) | 0.85 (0.68-1.06) | 1.23 (0.88-1.72) | 1.02 (0.64-1.62) | 0.82 (0.71-0.95) | 0.81 (0.58-1.13) | 0.54 (0.20-1.45) |
| med | 1.15 (0.98-1.36) | 1.09 (0.75-1.59) | 2.84 (1.93-4.19) | 0.86 (0.68-1.08) | 1.17 (0.81-1.70) | 2.02 (1.34-3.04) | 0.91 (0.78-1.06) | 1.09 (0.81-1.46) | 0.64 (0.26-1.57) |
| high | 0.98 (0.82-1.16) | 1.45 (1.07-1.97) | 1.29 (0.75-2.21) | 1.06 (0.86-1.32) | 0.94 (0.63-1.41) | 1.43 (0.94-2.19) | 1.00 (0.86-1.17) | 0.76 (0.56-1.04) | 0.67 (0.31-1.42) |
|  |  |  |  |  |  |  |  |  |  |

Model 4 - adjusted for: sex, age, and ethnicity, income, highest qualification, physical activity, sedentary behavior, total energy intake, body mass index, smoking status, and alcohol intake, total fat intake, fresh fruit intake, vegetables intake, total fibre intake, red meat intake and processed meat intake.

N number; HR hazard ratio; CI confidence interval
